# Should kyphoplasty curettes be used in nonosteoporotic patients? A cautionary tale

**DOI:** 10.1002/ccr3.2670

**Published:** 2020-01-30

**Authors:** Tobias A. Mattei, Caio M. Perret

**Affiliations:** ^1^ Division of Neurosurgery Saint Louis University Saint Louis MO USA; ^2^ Laboratory for Neuroprotection and Regenerative Strategies Federal University of Rio de Janeiro (UFRJ) Fundação Osvaldo Cruz (FioCruz) Rio de Janeiro Brazil

**Keywords:** balloon kyphoplasty, kyphoplasty curettes, osteoporotic fractures, vertebral fractures, vertebroplasty

## Abstract

The authors present the first report of a fracture of the tip of a kyphoplasty curette inside the vertebral body, which occurred during a procedure in a patient with non‐osteoporotic fracture. This highlights the need of further biomechanical research focused on the shear load failure properties of such type of pre‐bent curettes

## Graphical abstract

Further research on the shear load failure properties of prebent curettes as well as clinical studies are required before the use of such type of instrument can be recommended in nonosteoporotic patients.

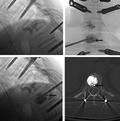

## 1 INTRODUCTION

Introduced in the early 2000s,^1^ kyphoplasty curettes have elicited significant interest among the spine surgery community, especially as an alternative for low‐pressure cement injection which may mitigate some of the possible associated concerns of balloon inflation in terms of worsening of the spinal canal compromise. This is especially relevant for fractures affecting the posterior wall of the vertebral body or in patients with tumoral lesions in the verrtebral body. Additionally, a recent biomechanical study revealed that using a curette before balloon kyphoplasty may reduce the rates of vertebral height loss after kyphoplasty, which could  have been related to a lack of cement interdigitation with the cancellous bone of the vertebral body adjacent to the cavity created by the balloon in the noncurette group.^2^ This study also demonstrated greater filling of the vertebral bodies by cement in the curette group, a difference that reached statistical significance.

A previous prospective randomized trial, which compared balloon kyphoplasty with kyphoplasty employing a curette followed by balloon inflation, demonstrated noninferiority of the latter in terms of vertebral body height restoration and similar complication rates in the treatment of vertebral compression fractures in patients with osteoporosis.^3^

Although the literature supporting the use of cement augmentation for traumatic fractures is less robust than that of osteoporotic vertebral fractures, such techniques have been extensively employed, either by themselves^4^ or in combination with minimally invasive screw and rod fixation^5^ for the treatment of thoracolumbar vertebral body fractures, especially those associated with extensive STIR changes in the vertebral body as demonstrated by MRI.

Up to now, there have been no reports of complications associated with the use of such curettes in traumatic/nonosteoporotic vertebral fractures in the literature. In the sequence, we present the first case report of a fracture of the tip of a kyphoplasty curette during a cavitational kyphoplasty for the treatment of a traumatic vertebral fracture.

## 2 CASE PRESENTATION

**Figure 1:**
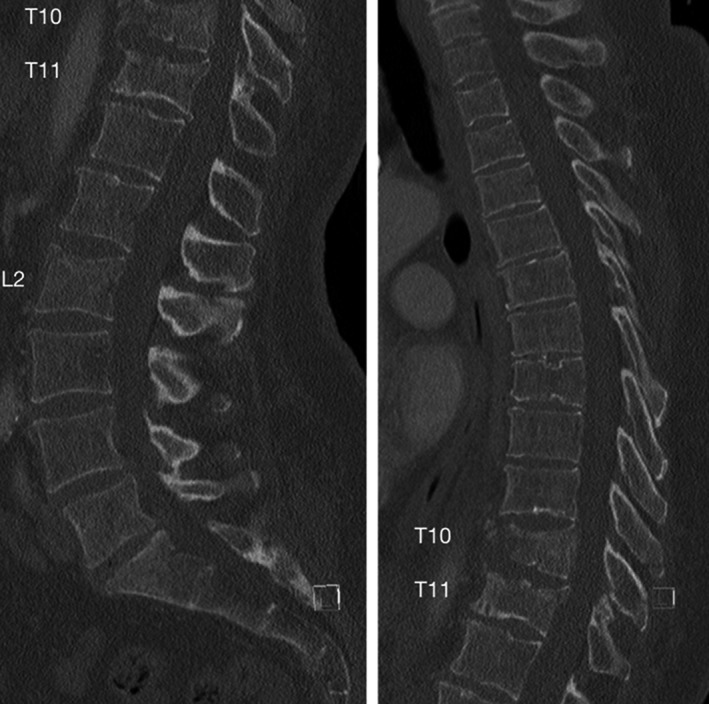
Preoperative CT‐scan demonstrating acute T10, T11, and L2 compression fractures with worse comminution at T10

**Figure 2:**
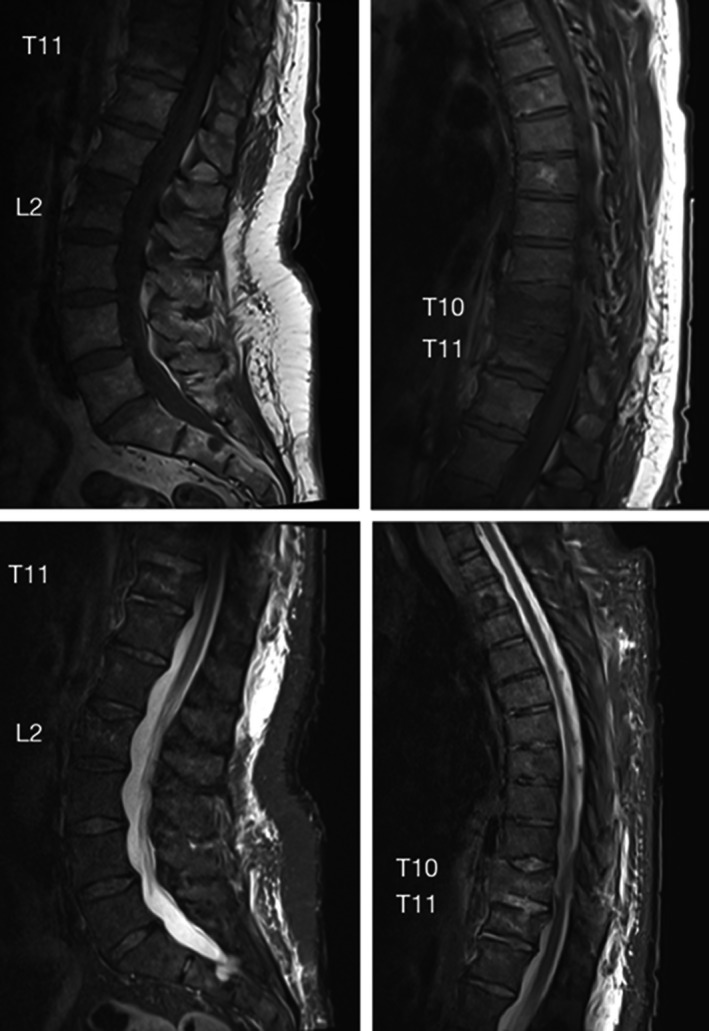
Preoperative MRI demonstrating some STIR changes at the T10, T11, and L2 vertebral bodies (bottom) with marked T1 hypo‐intensity in the same regions (top)

A 51‐year‐old female was brought to the hospital after a high‐energy motor vehicle accident which occurred after she lost control of her vehicle and fell into a ditch. The patient was neurologically intact but had extensive traumatic injuries including spleen laceration, possible aortic injury, and right femoral fractures. Additionally, in the screening CT‐scan of the abdomen, it was possible to visualize a T10 and T11 burst fractures as well as an L2 superior endplate fracture. Dedicated CT‐scan of the thoracic and lumbar spine confirmed the previous findings (Figure 1). MRI demonstrated the acute nature of the fractures (with STIR hyper‐intensity and marked T1 hypo‐intensity), but without any significant central canal  compromise (Figure 2).

Despite the low TLICS score of the fracture, we have previously demonstrated that comminuted burst fractures (A3) may be associated with significant kyphotic deformity if treated non‐operatively, especially when considering fractures at two adjacent levels as in the reported scenario (namely T10 and T11).^6^ As demonstrated by Figure 1, the T10 fracture is associated with the almost complete destruction of the anterior one‐third of the vertebral body. Although some may argue that cement augmentation should be reserved for osteoporotic fractures, there is an extensive literature, albeit mostly of retrospective nature, supporting the role of such therapeutic alternative in traumatic fractures.^4^, ^5^

**Figure 3:**
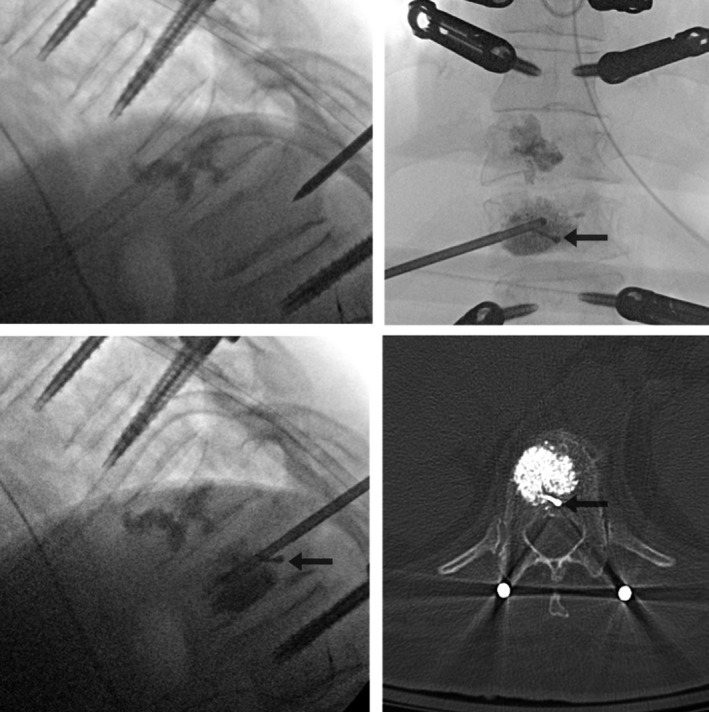
Intraoperative fluoroscopy after minimally invasive placement of pedicle screws T8‐L1. Left top: the Jamshidi needle is being advanced into the T11 vertebral body. Right top and left bottom: AP and lateral fluoroscopy, respectively, demonstrating the broken distal tip of the kyphoplasty curette. Bottom right: Axial slice of CT‐scan at the level of T11 vertebral body showing the broken tip of the kyphoplasty curette

**Figure 4:**
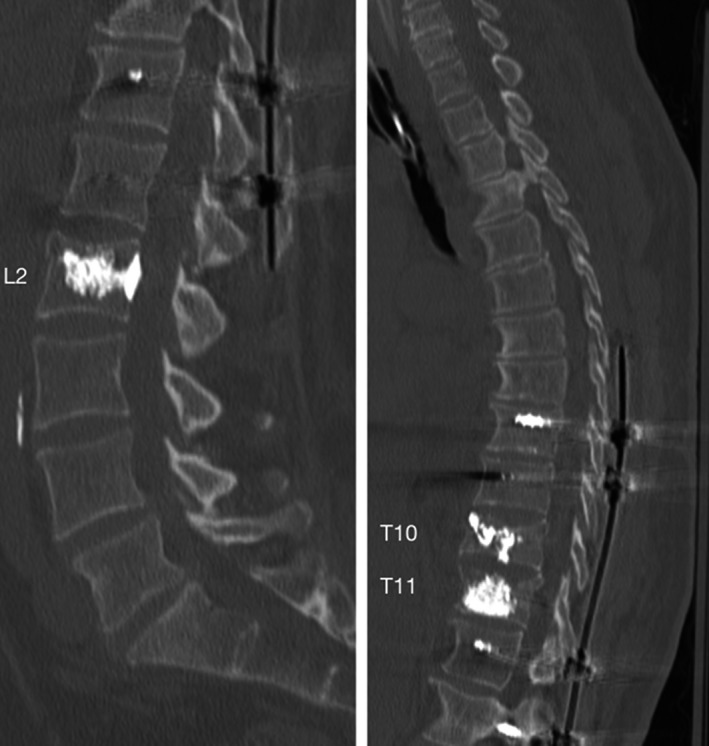
Postoperative CT‐scan demonstrating adequate placement of percutaneous screws and satisfactory filling of the T10, T11 and L2 vertebral bodies by methyl methacrylate

Ultimately, because of the comminuted pattern of the T10 burst fracture and the multilevel nature of the injuries, the patient was offered the option of proceeding with a minimally invasive navigation‐guided T8 to L1 posterior screw and rod fixation and T10, T11, and L2 kyphoplasties. After placement of the screws, the kyphoplasties were conducted (Figure 3). A stab incision was performed approximately 4.5 cm to the right of the midline at the levels of T10 and T11. Under direct AP and lateral fluoroscopy, Jamshidi needles were inserted through the skin and progressed until reaching the posterior portion of the vertebral bodies. Then, the inner trocars of the Jamshidi needles were removed. The cavity creator was inserted initially at T10, and moved forward and backward several times under direct live fluoroscopy in order to create a cavity and remodel the vertebral body. At the level of T11, after initial progression of the kyphoplasty curette beyond the tip of the Jamshidi needle a significant resistance was felt. While attempting to overcome such a resistance, lateral X‐rays demonstrated an excessive bending of the curved curette beyond its normal shape, although no fracture was observed yet. After the knob was rotated counterclockwise in order to bring the curette back inside its shaft, a sudden loss of resistance was felt. New X‐rays demonstrated a fracture of the distal portion of the kyphoplasty curette. Cement (PMMA) injection was conducted as planned fashion in T10, T11 and L2.  Final X‐rays demonstrated adequate placement of instrumentation and filling of the vertebral bodies by the cement. Postoperative CT‐scan confirmed the presence of broken curette tip in the T11 vertebral body (Figures 3 and 4). The patient presented progressive improvement of her axial mechanical low‐back pain and no complications at the follow‐up 1 year follow‐up.

## 3 DISCUSSION

Despite the lack of high‐quality scientific evidence on the topic, several retrospective series with a considerable number of patients have reported the benefits of cement augmentation of the fractured vertebra in addition to posterior screw and rod fixation, possibly allowing the use of short‐segment instrumentation for thoracolumbar burst fractures (i.e., A3 fractures according to the AO‐classification). 7, 8, 9Although a prospective comparative analysis between the clinical and radiological outcomes of such a combined strategy with kyphoplasty alone and posterior instrumentation without cement augmentation is still lacking in the literature, retrospective series have demonstrated that cement‐augmentation in associated with posterior instrumentation is associated with a significant improvement in clinical outcome measures (such as ODI, VAS, and SF‐36) as well as in radiological parameters (such as the mean kyphotic angle), without a significant increase in the risk of perioperative complications.^10^, ^11^ The restoration of the anterior column support has several advantages including providing immediate biomechanical stability and improving pain. Additionally, the restoration of anterior column support may reduce the rates of hardware failure, especially when considering minimally invasive percutaneous posterior screw and rod fixation, a technique in which, despite not achieving posterolateral fusion, has demonstrated several advantages in terms of decreasing perioperative complications especially in elderly and fragile patients.^11^

It has already been demonstrated by several different studies^12^, ^13^ that one of the reasons why kyphoplasty seems to be associated with lower rates of cement extravasation to endplates, epidural vessels and venous affluents of the inferior vena cava is the fact that the cement injection occurs at lower pressures once a cavity is created by the inflation of the balloon. In fact, it has been estimated that the injection pressure is reduced by more than threefold by the creation of a cavity in the vertebral body before cement injection (average maximum intravertebral pressure in the group with void creation versus the one without void creation of 1.20 vs 5.09 kPa, *P* = .001).^14^

Despite some arguments to the opposite,^15^ most spine surgeons would still consider a breach in the posterior wall of the vertebral body, especially if considering a pathological fracture with an associated tumoral lesion, at least a relative contraindication to kyphoplasty due to the fear of cement leakage toward the spinal canal as well as further posterior displacement of tumoral tissue and/or cortical bone with risk of new onset of neurological deficits.^16^

The use of curettes during kyphoplasty (a procedure to which we refer as cavitational kyphoplasty)^17^ has emerged in the past few years as an interesting alternative for low‐pressure cement injection while mitigating the possible associated risks of balloon inflation.Furthermore, there has been a growing amount of evidence suggesting that unilateral cement augmentation may provide similar benefits to a bilateral approach in terms of clinical outcomes, with lower operative times and radiation exposure.^18^ However, considering unilateral procedures, the supposed benefits of kyphoplasty over vertebroplasty in terms of restoration of vertebral body height become more questionable, especially taking into account that an inflated unilateral balloon would cover only a small percentage of the total surface area adjacent to the vertebral endplate, consequently increasing the likelihood of either an endplate fracture or, in the best scenario, restoration of height only in a small portion of the vertebral body.

Additionally, cavity creation plays a central role in some new forms of cement augmentation, such as the so‐called radiofrequency‐targeted vertebral augmentation (RF‐TVA‐D, Fine Europe GmbH, Mannheim, DE),^19^ a strategy currently available in Europe but not FDA‐approved.

Despite the fact that the choice between vertebroplasty, kyphoplasty, or other variations of cement augmentation as well as the approach (unilateral versus bilateral) is ultimately dependent on each surgeon's experience and personal preference, based on the rationale above, cavitational kyphoplasty has become a quite interesting option for those who favor unilateral approaches but who would still prefer a low‐pressure injection even if no restoration of vertebral height is intended. Even for those who prefer to use a kyphoplasty balloon, the use of curettes before balloon inflation consists in an interesting technical alternative, being especially useful, according to some reports,^20^ in patients with better bone quality.

From the billing standpoint, since 2015 the CPT code defines kyphoplasty as “*percutaneous vertebral augmentation, including cavity creation (fracture reduction and bone biopsy included when performed) using mechanical device (eg kyphoplasty), unilateral or bilateral cannulation, inclusive of all image guidance*.” The broad semantic meaning harbored by the expression “cavity creation using a mechanical device” has enabled procedures involving cement augmentation after the use of kyphoplasty curettes, to receive formal recognition as kyphoplasty and, therefore, to be billed as such even when no balloon is employed..

**Figure 5:**
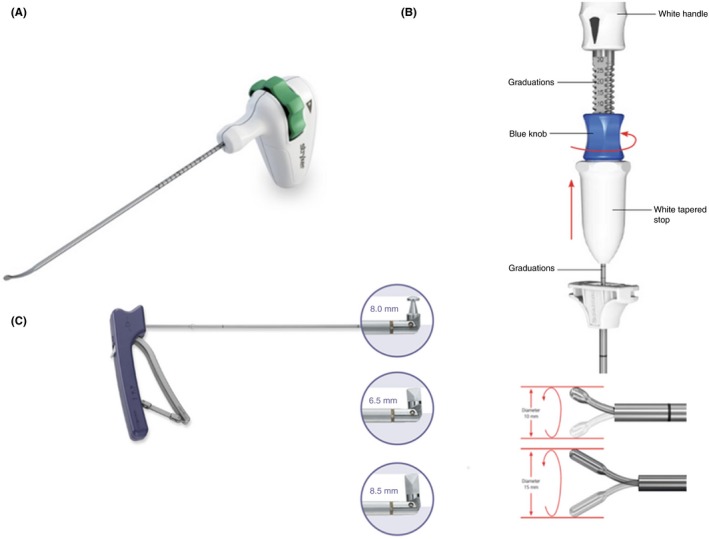
Available kyphoplasty curettes. A, iVAS balloon system/Stryker curette; B, Synflate™ Depuy‐Synthes kyphoplasty system curette; C, Medtronic Kyphon^®^ Express™ and Latitude II curette

There are currently two FDA‐approved types of curettes which have been used for cavitational kyphoplasty in the United States (Figure 5). The specific design of each one of these types of curettes involves significant differences which may affect their shear load failure points. The first type, which includes the iVAS balloon system/Stryker curette (Figure 3A) and the Synflate™ Depuy‐Synthes curette (Figure 3B), relies on a prebent curette which is straight when inside the cannulated shaft but which follows its normal curvature after the curette is outside the shaft. Depth control in these systems is performed through a knob located above the handle which advances the curette inside the shaft. The second design (KYPHON^®^ Express™ and Latitude II curette models/Medtronic) (Figure 3C) involves a separate articulation which is controlled by a trigger at the handle which can bend the tip of the curette from 180 to 90 degrees.

Each of these designs involves specific concerns regarding possible material failure. The prebent/angled curettes seem inherently vulnerable to shear forces associated with hard vertebral body bone which may cause bending of the curette beyond its normal curvature and lead to instrument failure at the junction of the straight and the bent portions of the curettes. Conversely, the separate articulation in the alternative design represents the weakest point of the system and, in the case of large shear forces, failure can be expected to occur at this articulation site. According to the manufacturer disclosures, the possibility of such event is reduced by a torque resetting device, which allows easy resetting of the T‐shaped tip when it encounters excess torsion.^21^

During the reported case, it was possible to observe a bent of the kyphoplasty curette right after its emergence from the cannula which was larger than expected, likely already a radiological sign of impending failure. Although it is unclear if there would be any different maneuver in order to retrieve the curette which would avoid the ultimate fracture, it should be highlighted that such type of excessive curvature may be an alert of imminent failure, after which any attempt to further advance the curette should be aborted.

In the presented situation, according to the manufacturer, the kyphoplasty curette was composed exclusively of titanium and therefore, its presence in the vertebral body should neither prevent obtaining further MRI if indicated nor have any other deleterious effects. In such a scenario, any surgical attempts to recover the lost fragment, which would ultimately involve the necessity of a corpectomy, seem quite unwarranted.

Up to now, there has been no report of instrument complication with the use of kyphoplasty curettes in traumatic fractures in nonosteoporotic patients, with some authors even advocating that such curettes may be an important adjuvant in the case of young patients with hard cancellous bone.^22^ Additionally, it has also been speculated that the use of a curette before balloon inflation may possibly reduce the risk of balloon rupture once the bone bridges and spikes are cleared by such instrument.^23^

This relative lack of information on the failure stress of such relatively  new instruments in the rapidly progressing field of kyphoplasty techniques (and the concomitant need of further biomechanical studies on the mechanical properties of such devices) is further corroborated by a similar recent report of an unretrievable curved kyphoplasty needle.^24^

## 4 CONCLUSIONS

Despite the increasing use of kyphoplasty curettes for cement augmentation, the literature on the safety of such instruments for the treatment of traumatic fractures in nonosteoporotic patients is quite limited. This case report serves as a cautionary tale about the possibility of instrument fracture, especially when employing prebent curettes for the treatment of traumatic vertebral fractures. Despite the anedoctic nature of the evidence provided by this short report, we believe it plays an important role insofar as it highlights the necessity of further research on the biomechanical properties of such tools, with special focus on the shear load failure of prebent curettes. Ultimately, we believe that further clinical studies are required before the use of such type of instrument can be recommended in the scenario of traumatic/nonosteoporotic fractures.

## CONFLICT OF INTEREST

The author declares that the article content was composed in the absence of any commercial or financial relationships that could be construed as a potential conflict of interests.

## AUTHOR CONTRIBUTIONS

TAM: Study design, Data collection, Manuscript composition, Review of the final version of the manuscript. CMP: Assistance with reference list, Figures adjustments, Review of the final version of the manuscript.

## References

[1] Vallejo R , Benyamin R , Floyd B , Casto JM , Joseph NJ , Mekhail N . Percutaneous cement injection into a created cavity for the treatment of vertebral body fracture: preliminary results of a new vertebroplasty technique. Clin J Pain. 2006;22:182‐189. 16428953 10.1097/01.ajp.0000169675.41815.49

[2] Alamin T , Kleimeyer JP , Woodall JR , Agarwal V , Don A , Lindsey D . Improved biomechanics of two alternative kyphoplasty cementation methods limit vertebral recollapse. J Orthop Res. 2018;36:3225‐3230. 30117192 10.1002/jor.24127

[3] Bastian L , Schils F , Tillman JB , Fueredi G , SCORE Investigators . A randomized trial comparing 2 techniques of balloon kyphoplasty and curette use for obtaining vertebral body height restoration and angular‐deformity correction in vertebral compression fractures due to osteoporosis. AJNR Am J Neuroradiol. 2013;34:666‐675. 23179647 10.3174/ajnr.A3363 PMC7964904

[4] Grelat M , Madkouri R , Comby PO , Fahed E , Lemogne B , Thouant P . Mid‐term clinical and radiological outcomes after kyphoplasty in the treatment of thoracolumbar traumatic vertebral compression fractures. World Neurosurg. 2018;115:e386‐e392. 29678706 10.1016/j.wneu.2018.04.060

[5] Hoppe S , Aghayev E , Ahmad S , Keel MJB , Ecker TM , Deml M , et al. Short Posterior Stabilization in Combination With Cement Augmentation for the Treatment of Thoracolumbar Fractures and the Effects of Implant Removal. Global Spine J. 2017;7:317–324. 28815159 10.1177/2192568217699185 PMC5546680

[6] Mattei TA , Hanovnikian J , Dinh HD . Progressive kyphotic deformity in comminuted burst fractures treated non-operatively: the Achilles tendon of the Thoracolumbar Injury Classification and Severity Score (TLICS). Eur Spine J. 2014;23(11):2255–2262. 10.1007/s00586-014-3312-0 24823845

[7] Kao FC , Hsieh MK , Yu CW , et al. Additional vertebral augmentation with posterior instrumentation for unstable thoracolumbar burst fractures. Injury. 2017;48:1806–1812. 28662833 10.1016/j.injury.2017.06.015

[8] Korovessis P , Repantis T , Petsinis G , Iliopoulos P , Hadjipavlou A . Direct reduction of thoracolumbar burst fractures by means of balloon kyphoplasty with calcium phosphate and stabilization with pedicle‐screw instrumentation and fusion. Spine (Phila Pa 1976). 2008;33(4):E100‐E108. 18277858 10.1097/BRS.0b013e3181646b07

[9] Afzal S , Akbar S , Dhar SA . Short segment pedicle screw instrumentation and augmentation vertebroplasty in lumbar burst fractures: an experience. Eur Spine J. 2008;17:336‐341. 18193300 10.1007/s00586-008-0587-z PMC2270394

[10] Marco RA , Kushwaha VP . Thoracolumbar burst fractures treated with posterior decompression and pedicle screw instrumentation supplemented with balloon‐assisted vertebroplasty and calcium phosphate reconstruction. J Bone Joint Surg Am. 2009;91:20‐28. 19122075 10.2106/JBJS.G.01668

[11] Krüger A , Rammler K , Ziring E , Zettl R , Ruchholtz S , Frangen TM . Percutaneous minimally invasive instrumentation for traumatic thoracic and lumbar fractures: a prospective analysis. Acta Orthop Belg. 2012;78:376‐381. 22822580

[12] Zhan Y , Jiang J , Liao H , Tan H , Yang K . Risk Factors for Cement Leakage After Vertebroplasty or Kyphoplasty: A Meta-Analysis of Published Evidence. World Neurosurg. 2017;101:633–642. 28192270 10.1016/j.wneu.2017.01.124

[13] Phillips FM , Todd Wetzel F , Lieberman I , Campbell-Hupp M . An *in vivo* comparison of the potential for extravertebral cement leak after vertebroplasty and kyphoplasty. Spine (Phila Pa 1976). 2002;27:2173–2178; discussion 2178–9. 12394934 10.1097/00007632-200210010-00018

[14] Li K , Yan J , Yang Q , Li Z , Li J . The effect of void creation prior to vertebroplasty on intravertebral pressure and cementdistribution in cadaveric spines with simulated metastases. J Orthop Surg Res. 2015;10:20. 25626462 10.1186/s13018-015-0160-5 PMC4338624

[15] Molloy S , Sewell MD , Platinum J , Patel A , Selvadurai S , Hargunani R , Kyriakou C . Is balloon kyphoplasty safe and effective for cancer-related vertebral compression fractures with posterior vertebral body wall defects? J Surg Oncol. 2016;113:835–842. 26996273 10.1002/jso.24222

[16] Denaro V , Longo UG , Maffulli N , Denaro L . Vertebroplasty and kyphoplasty. Clin Cases Miner Bone Metab. 2009;6:125–130. 22461161 PMC2781232

[17] Mattei TA . Cavitational kyphoplasty: a new technique for reducing the rates of cement extravasation through targeted low-pressure cement injection. Acta Neurochir (Wien). 2017;159:1153–1157. 28382398 10.1007/s00701-017-3167-3

[18] Sun H , Lu PP , Liu YJ , et al. Can unilateral kyphoplasty replace bilateral kyphoplasty in treatment of osteoporotic vertebral compression fractures? A systematic review and meta‐analysis. Pain Physician. 2016;1:551‐563. 27906934

[19] Dalton BE , Kohm AC , Miller LE , Block JE , Poser RD . Radiofrequency‐targeted vertebral augmentation versus traditional balloon kyphoplasty: radiographic and morphologic outcomes of an ex vivo biomechanical pilot study. Clin Interv Aging. 2012;7:525‐531. 23204845 10.2147/CIA.S37025 PMC3508556

[20] Teyssédou S , Saget M , Pries P . Kyphopasty and vertebroplasty. Orthop Traumatol Surg Res. 2014;100:S169‐S179. 24406028 10.1016/j.otsr.2013.11.005

[21] Medtronic announces new curette for treatment of vertebral compression fractures. http://phx.corporate-ir.net/External.File?t=2%26item=g7rqBLVLuv81UAmrh20Mp39r4ByBqUcSeqxbt8umC81M9r0wsvCWifDQEoY1OQKgewl5s0r20koUOUKYIr9j7g==. Accessed on 02.20.19.

[22] Becker S . The Technique of Balloon Kyphoplasty In: Becker S, Ogon M, (Eds.). Balllon Kyphoplasty. New York: Springer Wien; 2008; 49-70.

[23] Stevenson M , Gomersall T , Lloyd Jones M , et al. Percutaneous vertebroplasty and percutaneous balloon kyphoplasty for the treatment of osteoporotic vertebral fractures: a systematic review and cost‐effectiveness analysis. Health Technol Assess. 2014;18:1‐290. 10.3310/hta18170 PMC4780995 24650687

[24] Shah NA , Catlin E , Jassal N , Hafez O , Padalia D . Retained Curved Needle After Balloon Kyphoplasty: A Complication with a Novel Device and Its Management. Cureus. 2019;11:e4367. 31192072 10.7759/cureus.4367 PMC6551195

